# Pulmonary Nontuberculous Mycobacterial Disease, Ontario, Canada, 1998–2010

**DOI:** 10.3201/eid1911.130737

**Published:** 2013-11

**Authors:** Theodore K. Marras, David Mendelson, Alex Marchand-Austin, Kevin May, Frances B. Jamieson

**Affiliations:** University Health Network, Toronto, Ontario, Canada (T.K. Marras, D. Mendelson);; Mount Sinai Hospital, Toronto (T.K. Marras, D. Mendelson);; University of Toronto, Toronto (T.K. Marras, D. Mendelson, A. Marchand-Austin, F.B. Jamieson);; Public Health Ontario, Toronto (A. Marchand-Austin, K. May, F.B. Jamieson)

**Keywords:** atypical mycobacteria, atypical Mycobacterium, epidemiology, Mycobacterium avium-intracellulare, Mycobacterium avium-intracellulare complex, mycobacterium intracellulare, nontuberculous Mycobacterium, Canada, Ontario, tuberculosis and other mycobacteria, bacteria

## Abstract

We measured the prevalence and temporal trends of pulmonary nontuberculous mycobacterial disease among residents of Ontario, Canada, during 1998–2010. Five-year prevalence increased from 29.3 cases/100,000 persons in 1998–2002 to 41.3/100,000 in 2006–2010 (p<0.0001). Improved laboratory methods did not explain this increase, suggesting a surge in disease prevalence.

Pulmonary nontuberculous mycobacterial (pNTM) disease is clinically challenging. Therapy entails complex antimycobacterial drug combinations, typically for 18 months (*1*), often with poor tolerability (*2*) and limited success (*3*). pNTM disease is increasingly common in Canada (*4*) and the United States (*5*–*7*), but its prevalence is not well understood. Determining the epidemiology of pNTM disease is difficult for several reasons. It is generally not reportable, so population-level data are not routinely compiled. The diagnosis requires clinical and radiologic information in addition to microbiological examination (>2 positive sputum cultures or 1 bronchoscopic or biopsy culture) (*1*). Finally, the chronic nature of pNTM disease dictates longitudinal study, illustrated by considering that only a minority with pNTM disease appear to be treated (18% in 1 study) (*6*), treatment succeeds in only 56% (*3*), and disease recurs in >30% of patients (*2*,*8*). These data indicate that most pNTM cases are expected to be chronic. Cases detected by isolation of nontuberculous *Mycobacterium* spp. in 1 year, generally remain prevalent over several subsequent years, regardless of the reliable appearance of subsequent isolates, with a disease duration that may depend primarily on patient survival.

The traditional method of identifying cases for NTM disease epidemiology studies by using mycobacterial laboratory databases and measuring annual prevalence is not ideal. Such studies assume that, in patients with pNTM disease, the organism is isolated during every year of disease, an invalid assumption (*6*). Recent investigators have focused on prevalence within a defined period (period prevalence) as an improved estimate of pNTM disease, including a 2-year study in Oregon (*5*), 3-year sampling of 4 US health care delivery systems (*6*), and <11-year US-wide sample of Medicare beneficiaries (*7*). Important limitations of these studies included the patient populations and geographic regions selected and the limited data about temporal prevalence changes. Expanding on methods of previous studies to overcome some prior limitations, we performed a population-based study of pNTM disease in Ontario, Canada, using 5-year periods for prevalence calculations and compared prevalence from 1998–2002 to 2006–2010.

## The Study

We performed a retrospective cohort study of all Ontario residents who had pulmonary nontuberculous *Mycobacterium* spp. isolated during 1998–2010, identified from the records of the Public Health Ontario Laboratory, capturing ≈95% of NTM disease in Ontario. Culture was performed by using Bactec 460 TB system until 2000 and thereafter with BACTEC MGIT 960 (Becton Dickinson, Baltimore, MD, USA). Before 2008, speciation was performed by using a combination of DNA probes (AccuProbe, Gen-Probe, San Diego, CA, USA) for *Mycobacterium avium* complex (MAC) and *M. gordonae* and high-performance liquid chromatography for other species and thereafter solely by DNA probes (AccuProbe, Gen-Probe) or line-probe assays (GenoType, Hain Lifescience, Germany). Because MAC was not identified to individual species for most of our study, we present data only for MAC.

Full criteria for pNTM disease include the presence of all clinical (symptoms and radiology) and microbiological components (*1*). We defined surrogate criteria as microbiological criteria only (*1*), (>2 positive sputum cultures or 1 bronchoscopic or lung biopsy culture), which has a positive predictive value of 70%–100% (*5*,*6*,*9*,*10*). Period prevalence of disease was calculated as the number of persons who fulfilled the disease criteria during a 5-year period (1998–2002 or 2006–2010), divided by the Ontario population at the period midpoint. We left a 3-year gap (2003–2005) between periods to minimize patient overlap. We excluded *M. gordonae* from period prevalence because it is rarely pathogenic (*1*). We selected a conservative 5-year period on the assumption that the median survival with pNTM disease is 5–10 years (*10*,*11*), using the low end of the survival range based on assumptions that a small proportion of the cohort would not have true disease (misclassified by surrogate definition) and disease of an additional small proportion would be cured.

Annual isolation prevalence (number of persons in a calendar year with >1 pulmonary *Mycobacterium* spp. isolate divided by the contemporary population) and annual disease prevalence (number of persons in a calendar year whose illnesses fulfilled criteria for disease divided by the contemporary population) are presented for illustrative purposes. A generalized linear model with negative binomial distribution was used to assess annual rate changes, and a simple model binomial approach was used to compare 5-year period prevalence rates by using SAS 9.2 (SAS Institute, Cary, NC, USA). This study was approved by the University of Toronto Research Ethics Board with the requirement for informed consent waived.

Ontario’s population increased from 11.3 million to 13.2 million during 1998–2010. Total annual isolations of pulmonary *Mycobacterium* spp. rose from 11.4 to 22.2 per 100,000 persons (p = 0.0025, mean annual increase 6.3%) (Table 1; Figure). The relative frequency of different nontuberculous *Mycobacterium* isolates remained constant. The most common pulmonary nontuberculous *Mycobacterum* isolates in 2010 were MAC (12.2 isolations/100,000 persons), *M. xenopi* (3.9/100,000), *M. gordonae* (3.0/100,000), *M. fortuitum* (0.8/100,000), and *M. abscessus* (0.6/100,000). Among patients with different *Mycobacterium* spp. isolates in 2010, the following proportions were judged to have disease: MAC, 52%; *M. abscessus*, 50%; *M. xenopi*, 38%; and other non–*M. gordonae* species, 38%. Annual prevalence for all NTM disease combined rose from 4.9 cases to 9.8 cases per 100,000 persons (p<0.0001, mean annual increase 6.5%) (Table 1; Figure). Five-year prevalence of pNTM disease (*M. gordonae* excluded) increased from 29.3 cases per 100,000 persons in 1998–2002 to 41.3 per 100,000 in 2006–2010 (p<0.0001) (Table 2).

**Table 1:** Annual prevalence of all pulmonary nontuberculous mycobacterial disease, Ontario, Canada, 1998–2010*

Year	Isolation prevalence†	Disease prevalence‡
1998	11.4	4.9
1999	14.3	6.3
2000	15.1	6.1
2001	18.7	7.6
2002	21.0	8.1
2003	18.9	7.3
2004	22.8	8.6
2005	22.6	9.1
2006	23.4	9.7
2007	24.0	10.3
2008	24.5	10.4
2009	24.9	10.7
2010	22.2	9.8

*Annual (1-year) prevalence, per 100,000 population, in a calendar year. †Prevalence of >1 pulmonary nontuberculous *Mycobacterium* isolate. Mean annual increase: 6.3% (p = 0.025). ‡Prevalence of >2 sputum nontuberculous *Mycobacterium* isolates or 1 bronchoscopic or biopsy nontuberculous *Mycobacterium* isolate. Mean annual increase: 6.5% (p<0.0001).

**Figure:**
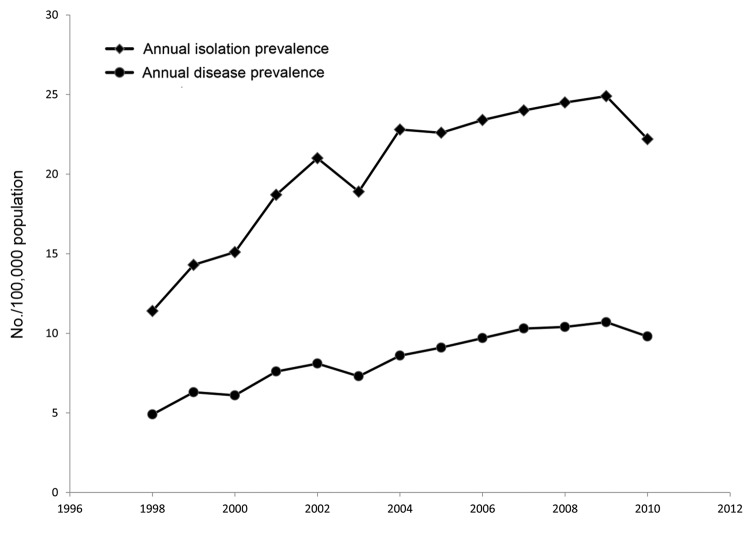
Annual isolation prevalence and disease prevalence per 100,000 persons of pulmonary nontuberculous mycobacteria, Ontario, Canada, 1998–2010.

**Table 2:** Five-year prevalence of pulmonarynontuberculous mycobacterial disease, Ontario, Canada, 1998–2002 and 2006–2010*

Isolate	1998–2002	2006–2010	p value
*M. avium* complex	18.0	26.5	<0.0001
*M. xenopi*	7.4	9.5	<0.0001
*M. fortuitum*	0.63	1.2	0.01
*M. abscessus*	0.63	1.2	<0.0001
Other nontuberculous *Mycobacterium* spp.	1.8	3.0	<0.0001
All nontuberculous *Mycobacterium* spp.	29.3	41.3	<0.0001

*Period prevalence of disease excludes *Mycobacterium gordonae* (a rarely pathogenic species) and is calculated as the total number of persons whose illness fulfilled disease criteria (>2 sputum nontuberculous *Mycobacterium* isolates or 1 bronchoscopic or biopsy nontuberculous *Mycobacterium* isolate) during 1 of the 5-year periods of interest (1998–2002 and 2006–2010), divided by the Ontario population at the midpoints of the periods (2000 for 1998–2002 and 2008 for 2006–2010).

## Conclusions

The 5-year prevalence of pNTM disease was substantial and increased significantly during our population-based assessment in Ontario, Canada. Our measurements of period prevalence (29.3 and 41.3 cases/100,000 persons) were substantially higher than observed in Oregon (8.6/100.000), probably partially because of the shorter period (2 years) and more stringent definition for disease (medical records review) used in the Oregon study (*5*). Other studies did not present period prevalence for the entire study populations, only by age strata, and used durations of 3 years (*6*,*7*) or <11 years (*7*). We selected a 5-year period assuming it would provide the most accurate estimate of disease prevalence based on the chronic nature of pNTM disease. Prior studies provided age-stratified data, with high period prevalence in older patients (20.4/100,000 to >200/100,000, depending on period length and specific age range) (*5*–*7*), as expected, because pNTM disease is a disease of the elderly (*1*,*4*,*6*,*12*). Although age data were unavailable for our study, annual disease prevalence of pulmonary MAC in Ontario has a strong age association, with an average increase of 14/100,000 per decade increase during 50–80 years (*4*).

Changes in microbiological methods and the number of samples submitted annually did not account for the increases in pulmonary nontuberculous *Mycobacterium* isolation (*13*). The attenuation in the rate of increase in isolation prevalence around the middle of the study corresponded with a previously reported plateau in the annual number of specimens submitted (*13*). However, the annual isolation prevalence continued to rise, and the annual disease prevalence rose steadily throughout the study period. We suspect a multifactorial explanation for the increase in pNTM disease: an increase in susceptible hosts (aging, chronic lung disease) contributes (*4*); decades-old increases in water aerosol exposure could cause recent increases in pNTM disease, given the potential latency of pNTM disease; more computed tomographic scanning probably leads to sampling patients with previously unidentified abnormalities; and reduced tuberculosis, with an associated reduction in cross-immunity, may play a role. The latter is supported by observations of increased extrapulmonary NTM infection in children not vaccinated with *M. bovis* BCG (*14*,*15*). pNTM disease in Ontario is substantial and increased greatly from early (1998–2002) to recent (2006–2010) periods.
